# Prevalence of Anxiety and Depression among Nurses during the early phase of COVID-19: A meta-analysis

**DOI:** 10.12669/pjms.41.2.10828

**Published:** 2025-02

**Authors:** Khalil Ahmed Jatt, Erika Sivarajan Froelicher, Abel Jacobus Pienaar, Khairunnisa Aziz Dhamani

**Affiliations:** 1 Khalil Ahmed Jatt, PhD, Shifa College of Nursing, Shifa Tameer-e-Millat University, Islamabad, Pakistan; 2 Erika Sivarajan Froelicher, PhD Professor of Epidemiology & Biostatistics, School of Medicine, University of California San Francisco, California, United States; 3 Abel Jacobus Pienaar, PhD Professor, Boitekanelo College, Gaborone, Botswana. Adjunct Professor, Durban University of Technology, Faculty of Health Science, Durban, South Africa; 4 Khairunnisa Aziz Dhamani, PhD, Shifa College of Nursing, Shifa Tameer-e-Millat University, Islamabad, Pakistan

**Keywords:** Anxiety, COVID-19, Depression, Meta-analysis, Nurses, Pandemic, Review, Systematic

## Abstract

**Background & Objective:**

The COVID-19 pandemic exposed significant mental health challenges among healthcare workers, particularly nurses, who are key frontline responders and are the largest segment of the global health workforce. Given the ongoing threat of Mpox and potential new COVID-19 variants, understanding these challenges is vital. This review estimates the prevalence of anxiety and depression among nurses during the early phase of the pandemic to inform future pandemic responses.

**Methods:**

A systematic review was conducted to identify studies published from January 1st to November 9, 2020. The quality of the included studies was assessed using the JBI Critical Appraisal Checklist. This review was reported according to the Preferred Reporting Items for Systematic Reviews and Meta-Analyses guidelines. A meta-analysis was performed using a random-effects model to estimate the pooled prevalence of anxiety and depression.

**Findings:**

Twenty-seven studies, involving 39,386 nurses from ten countries, were included in the meta-analysis. The pooled prevalence of anxiety across 24 studies was 38.54% (95% CI: 33.99, 43.10) (I^2^ = 97.89%). The pooled prevalence of depression in 22 studies was found to be 35.52% (95% CI: 26.61, 44.43) (I^2^ = 99.72%).

**Interpretation:**

The pooled prevalence of anxiety and depression in nurses caring for patients with COVID-19 during the early phase of the pandemic was higher than that in other healthcare workers. With the ongoing Mpox outbreak and the potential for future pandemics, these findings necessitate timely screening and robust mental health strategies to support nurses and enhance healthcare resilience.

## BACKGROUND

The COVID-19 pandemic not only overwhelmed global healthcare systems but also brought to light the profound psychological toll on those at the forefront—our healthcare workers. Among these, nurses, who constitute the largest segment of the global health workforce (59%),^1^ faced the greatest impact of this unprecedented crisis. As the backbone of healthcare delivery, their mental well-being is essential for the overall functioning of health systems. However, as highlighted by Allsopp et al.,^2^ the psychological scars of such major disasters often run deeper and last longer than physical injuries, yet they are frequently underestimated in disaster planning and resource allocation. This oversight is especially concerning for nurses, particularly female nurses, who are disproportionately vulnerable to mental health challenges during crises.^3^,^4^

A systematic review and meta-analysis of 13 studies by Pappa et al.^5^ found that the pooled prevalence of anxiety and depression among healthcare workers, in general, during COVID-19 was 23.2% and 22.8%, respectively. The review, which included studies published up to April 17, 2020, provides early evidence of the prevalence of anxiety and depression in healthcare workers.

The ongoing threat of emerging infectious diseases, such as the recent Mpox (monkeypox) outbreaks and the potential emergence of new variants of COVID-19, accentuates the need to understand and address the mental health challenges faced by healthcare workers, particularly nurses. Nurses are frontline defenders in healthcare crises, and their mental well-being is crucial for maintaining the quality and effectiveness of healthcare delivery. This review provides an essential estimate of the prevalence of anxiety and depression among nurses during the early phase of the COVID-19 pandemic. By examining these mental health outcomes, this review aims to inform strategies for early detection, prevention, and management of psychological distress in nurses during future pandemics. Understanding these dynamics will help to fortify healthcare systems against the psychological toll of future health emergencies, ensuring that nurses remain resilient and effective in their critical roles.

For the purposes of this review, the “early phase” of the pandemic is conceptualized as the initial months of COVID-19’s global spread, prior to the widespread availability of standardized preventive measures, evidence-based treatment protocols, and established psychological support frameworks. This period was characterized by heightened uncertainty, evolving clinical guidelines, and rapidly shifting healthcare priorities.

### Question statement:

What was the prevalence of anxiety and depression among nurses caring for COVID-19 patients during the early phase of the pandemic?

## METHODS

This systematic review and meta-analysis were conducted using rigorous methods to ensure the reliability and validity of the findings. The reporting of this review adheres to the 2020 Preferred Reporting Items for Systematic Reviews and Meta-Analyses (PRISMA)^6^ guidelines, and the quality of included studies was evaluated using the JBI Critical Appraisal Checklist. A meta-analysis was performed using a random-effects model to estimate the pooled prevalence of anxiety and depression.

### Search Strategy:

To search for relevant literature, PubMed and Cumulative Index of Nursing and Allied Health Literature (CINAHL) were utilized as primary databases. World Health Organization’s COVID-19 database was also used to search for more literature. Furthermore, the citation search and similar article search options provided by the databases were used to find more literature. The search terms used were: (anxiety OR anxious OR anxiety symptoms OR depression OR depressive OR depressive symptoms OR depressed) AND (nurses OR nursing staff OR nurse OR caregiver OR health care professionals OR healthcare workers) AND (COVID-19 OR coronavirus OR 2019-ncov OR sars-cov-2 OR cov-19). The search was last performed on November 9, 2020. Search strategies and details of search results for PubMed and CINAHL have been provided in Supplements.

### Studies Selection Criteria:

Criteria for inclusion involved studies that detailed prevalence rates of anxiety and depression in nurses who care for patients suspected or confirmed to have COVID-19; reports of studies published in the English language; studies conducted on other healthcare workers were also included if the prevalence of anxiety or depression among nurses could be ascertained. Exclusion criteria included studies that did not present separate prevalence data exclusively for nurses, or those combining nurses with other professionals (e.g., student nurses, midwives) without distinct reporting. Assessment of the eligibility criteria was performed by the primary researcher.

### Data Extraction and Quality Assessment:

Two independent researchers conducted the data extraction and evaluated the quality of the studies. The Excel sheets were structured to include data on the percent and count of nurses exhibiting symptoms of depression and anxiety. Additionally, the following information was also obtained: first author; month and year of publication; country; study setting; period of data collection; study methods; sample size; gender of nurses; response rates; tools for measuring anxiety or depression; cut-off scores of tools; the prevalence of anxiety and depression in males and females; the prevalence of mild, moderate, and severe anxiety and depression prevalence; funding sources; conflict of interest; and DOI (digital object identifier); sources of articles. If required, necessary calculations were done (for example, conversions from numbers to percentages etc.). For missing information, supplementary files were accessed (n = 2) and data was obtained from one corresponding author (n = 1) out of the authors who were contacted for obtaining more data (n=3). Both researchers independently extracted data from the included reports using Excel sheets. Comments about important points were saved in the form of notes in the relevant cells of the Excel sheets by each researcher. The differences between the values in both sheets were discussed and errors were resolved by recalculating or extracting the data again. The data in the final Excel sheet was also verified multiple times.

The quality of studies was assessed through the latest version of the *JBI Critical Appraisal Checklist for Studies Reporting Prevalence Data*.^7^ The checklist was found to be a “valid approach to assessing the methodological quality of studies reporting prevalence data to be included in systematic reviews”.^8^ Both researchers independently performed the quality assessment of the studies using Excel sheets for the JBI checklist. The important points were saved in the form of notes in the relevant cells of the Excel sheets. The differences were discussed based on the saved notes and adjustments were made in the final sheet.

### Data Synthesis and Analysis:

The number of nurses screened positive (numerator) in the studies was retrieved along with the total number of nurses screened (denominator). Using this prevalence data, confidence intervals (CI) were calculated using a Microsoft Excel sheet provided by Neyeloff et al.^9^ Using percentage prevalence, sample size, and CIs, the pooled prevalence for anxiety and depression were calculated and forest plots were developed in Stata software (version 16). Because the study populations were considerably different from each other, we employed the DerSimonian and Laird random-effects model within Stata for the calculation of pooled prevalence.**10** Subgroup analysis for the kind of instruments used to measure anxiety was also included in the analysis using STATA.

## RESULTS

**Fig.1:**
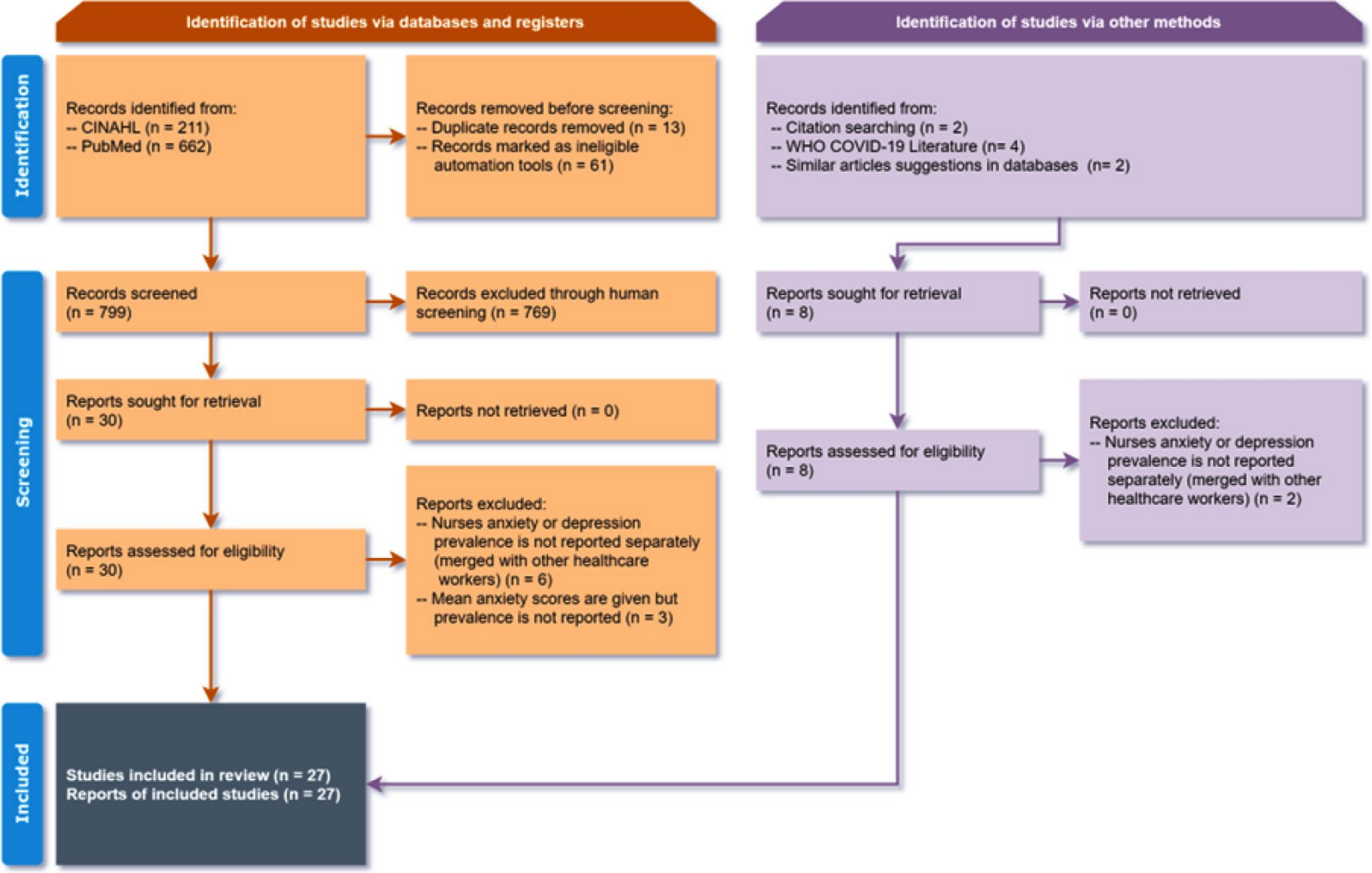
PRISMA 2020 Flow Diagram of the Selection Process of Studies.

Twenty-seven studies were included, were included, totalling 39,386 participants. Studies published from January 1 to November 9, 2020, were considered. The study retrieval and screening process, following the PRISMA framework, is shown in Fig.1.

### Salient Features of the Included Studies:

All studies were cross-sectional, with a median of 223 nurses per study (range: 17–22,034). In the 16 studies reporting gender, most nurses were female (32,102 [90.9%]). Fifteen studies^3^,^11^-^24^ with 37,033 participants were conducted in China, four in Turkey^25^-^28^ with 981 participants, and the remaining eight studies in Brazil^29^, India^30^, Iran^31^, Nepal^32^, the Philippines^33^, Saudi Arabia^34^, Singapore^35^, and South Korea.^36^ The instruments used to measure anxiety were: Generalized Anxiety Disorder 7-item (GAD-7), Zung Self-rating Anxiety Scale (SAS), Hospital Anxiety and Depression Scale (HAD), Hamilton Rating Scale for Anxiety (HAM-A), COVID-19 Anxiety Scale (CAS) and State-Trait Anxiety Inventory (STAI). For depression, the Patient Health Questionnaire (PHQ-9), Zung Self-Rating Depression Scale (SDS), Hospital Anxiety and Depression Scale (HAD), and Beck Depression Inventory (BDI) were used. Key study characteristics and findings are summarized in Table-I.

**Table-I:** Key characteristics and findings of the included studies (N = 27).

Authors	Countries	*Month of data collection (2020)	Number of Nurses	Females % (n)	Response Rate	Tools for Anxiety	Cut-point	Prevalence Anxiety % (n)	Tools for Depression	Cut-off Scores	Prevalence Depression % (n)
Cao et al. 11	China	January	19	NR	100%	NA	NA	NA	PHQ-9	≥10	47.3 (9)
Lee et al. 35	Singapore	January – Feb.	155	NR	86%	HAD	≥11	19.2 (30)	HAD	≥11	18.1 (28)
Liu et al. 12	China	January – Feb.	2826	NR	NR	SAS	≥50	17.6 (497)	SDS	≥50	39.2 (1108)
Zhan et al. 13	China	January – Feb.	2667	97 (2586)	96%	GAD-7	≥5	39.8 (1062)	PHQ-9	≥5	54.7 (1458)
Hu et al. 14	China	February	2014	87.1 1754)	78%	SAS	≥50	41.4 (833)	SDS	≥53	43.6 (878)
Ning et al. 3	China	February	295	98.0 (289)	NR	SAS	50	20.3 (60)	SDS	≥53	30.2 (89)
Şahin et al. 25	Turkey	February	254	NR	NR	GAD-7	≥5	66.5 (169)	PHQ-9	≥5	79.1 (201)
Xiong et al. 15	China	February	223	97.3 (217)	61.80%	GAD-7	≥5	40.8 (91)	PHQ-9	≥5	26.4 (59)
Yörük & Güler 26	Turkey	February	173	NR	NA	NA	NA	NA	BDI	≥17	26.6 (46)
Zheng et al.^16^	China	February	3228	96.7 (3121)	61%	SAS	≥50	18.1 (585)	SDS	≥50	34.3 (1107)
X. Yang et al. 17	China	February	164	NR	NR	SAS	≥50	36.0 (59	NA	NA	NA
AlAteeq et al. 34	Saudi Arabia	March	132	NR	NR	GAD-7	≥10	37.9 (50)	PHQ-9	≥10	32.9 (43)
An et al. 18	China	March	1103	90.7 (1001)	NR	NA	NA	NA	PHQ-9	≥5	43.6 (481)
Khanal et al. 32	Nepal	March	167	NR	NR	HAD	>7	56.2 (94)	HAD	>7	46.7 (78)
Dal’Bosco et al. 29	Brazil	March -April	88	89.8 (79)	NR	HAD	≥8	48.9 (43)	HAD	≥8	25.0 (22)
Pouralizadeh et al. 31	Iran	April	441	95 (420)	NR	GAD-7	≥10	38.8 (171)	PHQ-9	≥10	37.4 (165)
Aksoy & Koçak 27	Turkey	April	431	87.2 (376)	NR	STAI	≥41	89.5 (386)	NA	NA	NA
Han et al. 19	China	April	22034	98.6 (20909)	NR	SAS	≥50	20.6 (4539)	SDS	≥50	28.7 (6324)
Saricam 28	Turkey	April	123	74.0 (91)	NR	STAI	≥57	46.3 (57)	NA	NA	NA
Suryavanshi et al. 30	India	April	47	NR	NR	GAD-7	≥5	59.6 (28)	PHQ-9	≥5	61.7 (29)
Wang et al. 20	China	April	1334	NR	73.60%	GAD-7	≥7	28.6 (382)	PHQ-9	≥10	15.6 (208)
Zhu et al. 21	China	April	86	100 (86)	NR	SAS	≥50	27.9 (24)	SDS	≥50	43.0 (37)
Labrague & Santos 33	Philippines	April - May	325	74.8 (243)	93%	CAS	≥9	37.8 (123)	NA	NA	NA
Lai et al. 22	China	April - May	764	90.8 (694)	68%	GAD-7	≥7	12.7 (97)	PHQ-9	≥10	15.4 (118)
Tu et al. 23	China	April - May	100	100 (100)	100%	GAD-7	≥4	40 (40)	PHQ-9	≥4	46.0 (46)
S. Yang et al. 36	South Korea	May	17	NR	96%	GAD-7	≥5	58.8 (10)	PHQ-9	≥10	0 (0)
Li et al. 24	China	June	176	77.3 (136)	NR	HAM-A	NR	77.3 (136)	NA	NA	NA

NR = Not Reported, NA = Not Applicable

* The studies are sorted according to the month of data collection.

### Quality Assessment of the Studies:

A summary of the quality assessment using the JBI Critical Appraisal Checklist for Prevalence Studies^37^ is provided in Table-II, detailing responses to nine checklist questions.

**Table-II:** Summary of the Quality Assessment of the Studies.

Studies	Question Numbers of JBI Checklist and Assessment of the Studies								

	1	2	3	4	5	6	7	8	9
Aksoy & Koçak 27	?	_	+	+	+	+	+	+	?
AlAteeq et al. 34	–	–	+	+	+	+	+	+	?
An et al. 18	?	–	?	+	+	+	+	+	?
Cao et al. 11	+	+	+	+	+	+	+	+	+
Dal’Bosco et al. 29	+	+	+	+	+	+	+	+	?
Han et al. 19	?	–	+	+	+	+	+	+	?
Hu et al. 14	+	+	+	+	+	+	+	+	+
Khanal et al. 32	+	–	–	+	+	+	+	+	–
Labrague & Santos 33	+	+	+	+	+	+	+	+	+
Lai et al. 22	+	+	+	+	+	+	+	+	+
Lee et al. 35	+	+	+	?	+	+	+	+	+
Li et al. 24	?	–	?	+	+	+	+	+	?
Liu et al. 12	+	?	+	+	+	+	+	+	?
Ning et al. 3	?	–	?	+	+	+	+	?	?
Pouralizadeh et al. 31	+	–	?	+	+	+	+	+	?
S. Yang et al. 36	?	?	?	–	+	+	+	+	?
Şahin et al. 25	?	–	+	+	+	+	+	+	?
Saricam 28	?	–	?	+	+	+	+	+	?
Suryavanshi et al. 30	+	–	?	+	+	+	+	+	?
Tu et al. 23	?	?	?	–	+	+	+	+	+
Wang et al. 20	?	–	+	+	+	+	+	+	+
X. Yang et al. 17	?	–	?	+	+	+	+	+	?
Xiong et al. 15	+	+	–	+	+	+	+	+	+
Yörük & Güler 26	–	–	?	+	+	+	+	+	?
Zhan et al. 13	+	+	+	+	+	+	+	+	+
Zheng et al.^16^	+	–	?	+	+	+	+	+	+
Zhu et al. 21	–	?	?	+	+	+	+	+	?

**Key:**	Yes	+		No	–		Unclear	?

1. “Was the sample frame appropriate to address the target population?
2. Were study participants sampled in an appropriate way?
3. Was the sample size adequate?
4. Were the study subjects and the setting described in detail?
5. Was the data analysis conducted with sufficient coverage of the identified sample?
6. Were valid methods used for the identification of the condition?
7. Was the condition measured in a standard, reliable way for all participants?
8. Was there appropriate statistical analysis?
9. Was the response rate adequate, and if not, was the low response rate managed appropriately?”

### Prevalence of Anxiety:

**Fig.2:**
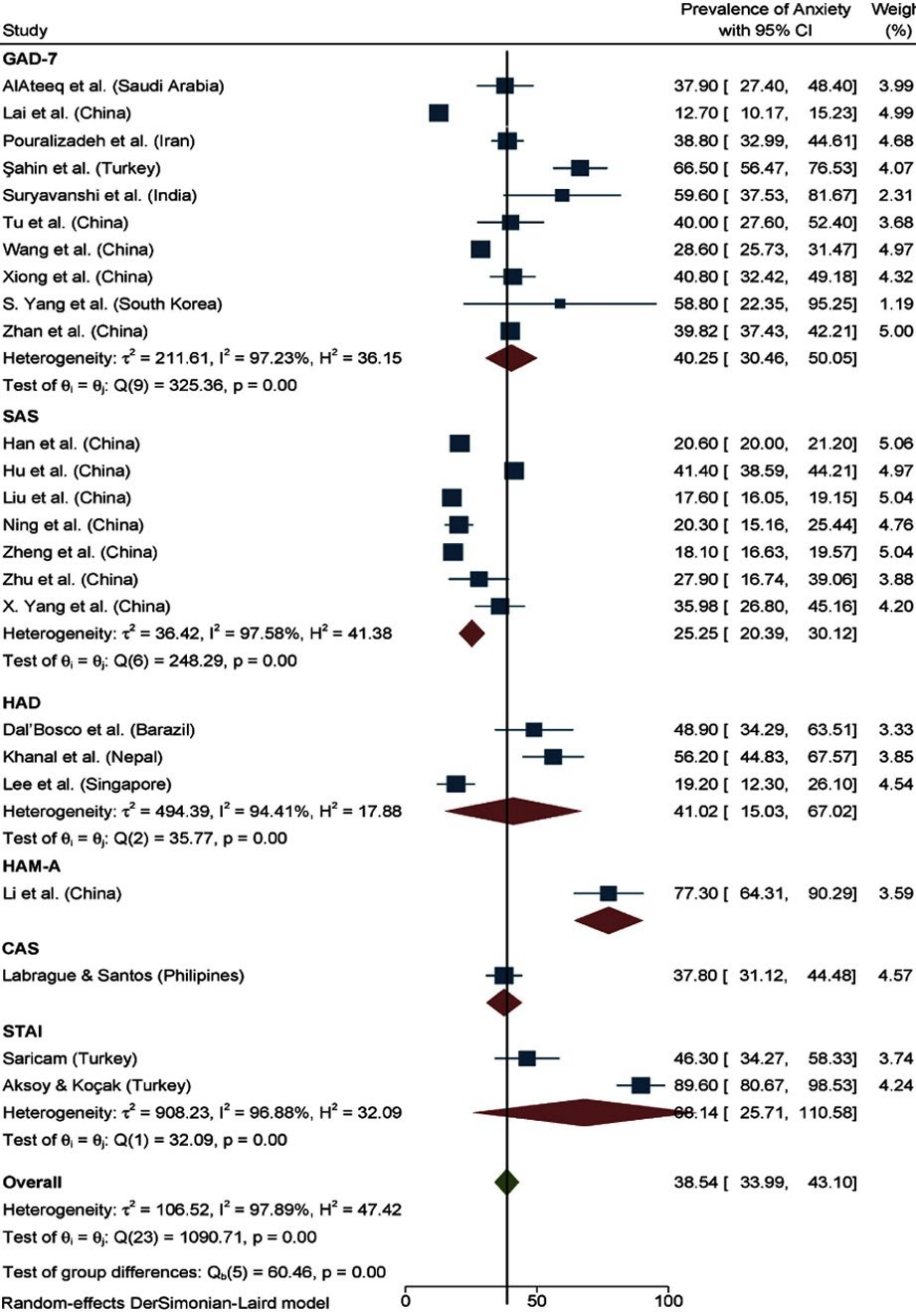
Forest Plot Presenting Pooled Prevalence of Anxiety by Instruments.

Anxiety was assessed in 24 of 27 studies, with a pooled prevalence of 38.54% (95% CI: 33.99, 43.10) (I² = 97.89%). Subgroup analysis by measurement tools showed that GAD-7 was used in 10 studies with a pooled prevalence of 40.25% (95% CI: 30.46, 50.05) (I² = 97.23%), SAS in six studies with 25.25% (95% CI: 20.39, 30.12) (I² = 97.90%), and HAD in three studies with 41.2% (95% CI: 15.03, 67.02) (I² = 94.41%). Fig.2 presents a forest plot of anxiety prevalence by instrument.

### Prevalence of Depression:

**Fig.3:**
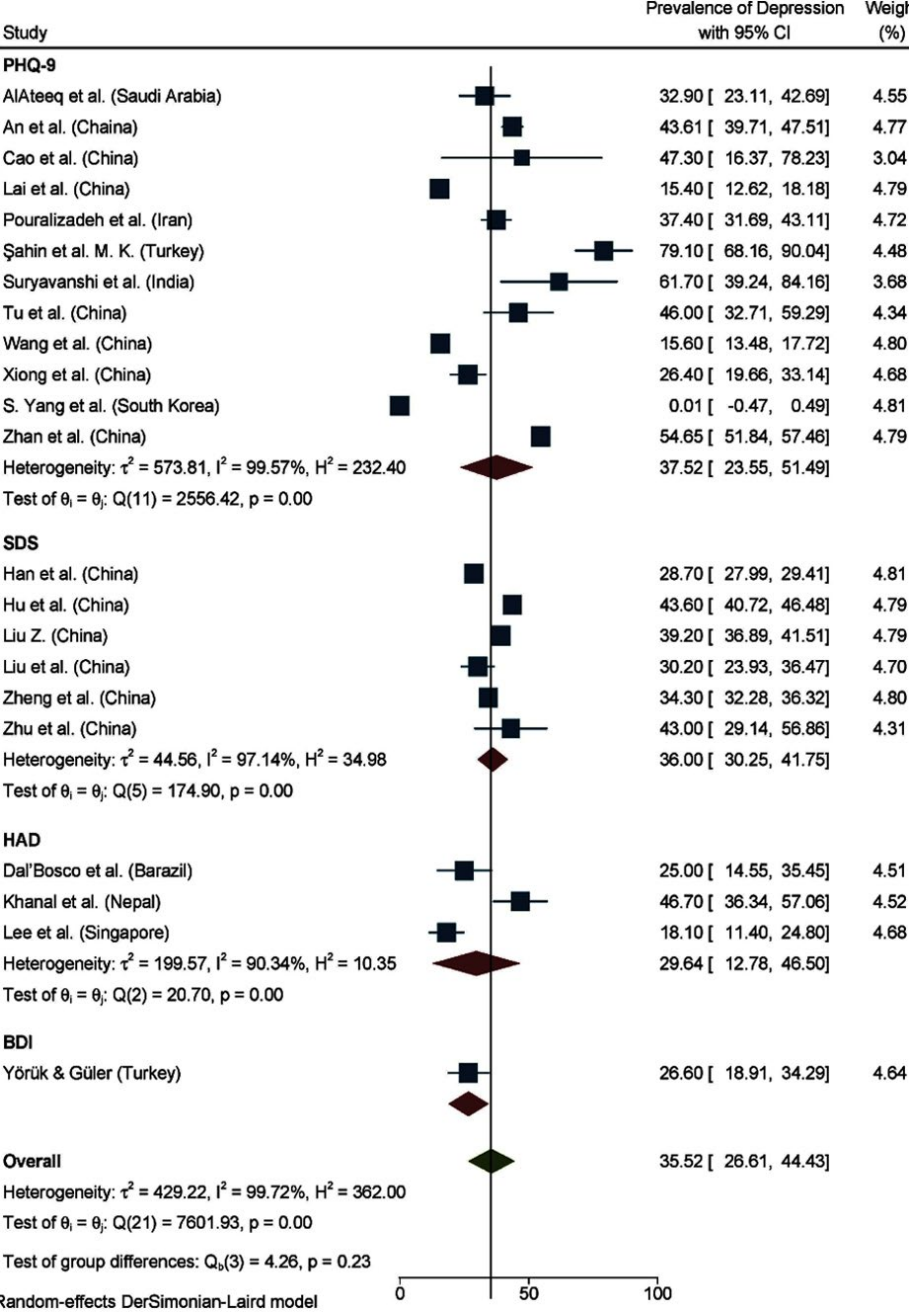
Forest Plot Presenting Pooled Prevalence of Depression by Instrument.

Depression was assessed in 22 of the 27 studies, with a pooled prevalence of 35.52% (95% CI: 26.61, 44.42) (I² = 99.72%). PHQ-9 was used in 12 studies with a pooled prevalence of 37.52% (95% CI: 23.55, 51.49) (I² = 99.57%), SDS in 6 studies with 36.00% (95% CI: 30.25, 41.75) (I² = 97.14%), and HAD in three studies with 29.64% (95% CI: 12.78, 46.50) (I² = 90.34%). Fig.3 shows a forest plot of depression prevalence by instrument.

## DISCUSSION

Our meta-analysis of 27 cross-sectional studies, involving 39,386 nurses, reveals a substantial prevalence of anxiety (38.54%) and depression (35.52%) among nurses caring for COVID-19 patients during the early phase of the pandemic. These rates are notably higher than those reported among healthcare workers in general (anxiety 23.21%, depression 22.8%) in an earlier meta-analysis.^5^ This stark difference emphasizes the unique psychological burden shouldered by nurses, who are not only the largest segment of the global health workforce but also the most directly engaged in patient care during such crises.

Our findings parallel those of Pappa et al.^5^, who also reported elevated anxiety and depression levels among healthcare workers early in the pandemic. While their meta-analysis encompassed a broader range of healthcare professionals, our review focuses specifically on nurses, revealing higher prevalence estimates. This comparison suggests that nurses, as front-line responders, may have borne a disproportionate psychological burden, emphasizing the need for targeted mental health interventions.

The heightened levels of anxiety and depression observed in nurses can be attributed to several factors. The early phase of the pandemic was characterized by an unprecedented surge in workload and patient contact, leading to significant emotional stress.^12^,^19^,^38^ This period also saw reports of suicide attempts among nurses,^39^,^40^ highlighting the need for further exploration into the causes of these mental health challenges and the potential somatization of psychological symptoms.^41^ The gender disparity in mental health outcomes is particularly concerning. Gender-specific data was not reported in all the studies (Table-I). However, limited data in our review, indicate that female nurses experienced higher rates of anxiety (51.53%) and depression (55.7%) compared to their male counterparts (27% and 17%, respectively).

These findings strongly suggest that nurses are more susceptible to mental health issues than other healthcare workers.^17^,^38^,^42^-^45^ Consequently, there is a pressing need for policymakers to recognize this vulnerability and implement targeted measures for the early detection and management of anxiety and depression among nurses, especially during future pandemics. While this review did not explore the root causes of nurses’ heightened susceptibility to mental health problems, future research should focus on this critical area.

Our analysis revealed high heterogeneity (I²) in the pooled prevalence of anxiety (I² = 97.89%) and depression (I² = 99.72%). This variability is likely due to the different cut-off points used across studies and the diverse populations studied. Despite this, the relatively narrow confidence intervals reported suggest that the pooled estimates are robust. However, it is crucial to interpret the I² statistic with caution, as it may not always indicate high heterogeneity in meta-analyses of point estimates.^46^,^47^

Our findings closely mirror those of Al Maqbali et al.,^48^ who reported anxiety at 37% and depression at 35% among nurses, emphasizing the importance of early mental health support during pandemics. Future research should focus on understanding the specific stressors and challenges that contribute to high rates of anxiety and depression among nurses during these crises. This knowledge can help hospital administrators provide psychological support services more effectively, including the use of telemedicine and informal support networks, particularly when resources are constrained.

### Strengths & Limitations of study:

This review is not without limitations. The potential for publication bias exists, as studies showing positive results are more likely to be published than those with negative findings. Additionally, the majority of included studies were conducted in China (n=15), raising concerns about the generalizability of our findings to nurses in other countries. Furthermore, while two researchers independently extracted data and assessed study quality, the literature search and screening for eligibility were performed by a single researcher. We did not conduct a sensitivity analysis, which could have provided further insights into the variability of our findings. Finally, there is some overlap among the populations studied, particularly in the Chinese studies, which may limit the independence of our results.

Despite these limitations, this meta-analysis is one of the first to report on the psychological toll of COVID-19 on nursing personnel during the early phase of the pandemic. The use of confidence intervals for our prevalence estimates adds precision to our findings. Additionally, all included studies met more than five criteria on the *JBI Critical Appraisal Checklist for Prevalence Studies*, further strengthening the validity of our results.

## CONCLUSION

Our systematic review and meta-analysis emphasize the significant prevalence of anxiety (38.54%) and depression (35.52%) among nurses during the early phase of the COVID-19 pandemic. These mental health challenges could potentially impair nurses’ performance and the quality of care provided. It is imperative that policymakers prioritize the early screening and management of these issues in nurses, particularly during pandemic-like events. Future research should delve into the underlying factors contributing to nurses’ susceptibility to mental health problems and explore innovative interventions such as telemedicine to provide timely psychological support. By addressing these challenges, healthcare systems can become more resilient, ensuring that nurses remain effective and supported in their vital roles during global health crises.

### Authors’ Contributions:

**KAJ:** Co-conceptualized the research study, conducted the systematic review, analyzed the data, and prepared the manuscript.

**ESF:** Co-conceptualized the research study, provided primary guidance in the methodology of the systematic review, supervised the analysis, and contributed to manuscript revisions. ESF is primarily responsible and accountable for the accuracy and integrity of the overall work.

**AJP** and **KAD:** Provided critical feedback and suggestions for improvement at all stages and contributed to manuscript editing and finalization.
